# Supplementary material for: Topological metal-insulator transition within the ferromagnetic state
Source: Nat Commun. 2026 Feb 27;17:2112. doi: 10.1038/s41467-026-70042-w (PMC12954081; doi:10.1038/s41467-026-70042-w)
Supplement: Supplementary file 1 — Supplementary Information [file 41467_2026_70042_MOESM1_ESM.pdf]

Supplementary materials (Topological Metal-Insulator Transition within the Ferromagnetic state)

(Dated: January 12, 2026)

CONTENTS

S1. Inelastic X-ray Scattering S2

S2. Neutron Scattering S3

A. Magnetic structure S3

B. Magnetic excitations S5

S3. Theoretical Methods S6

A. DFT Calculations S6

B. Construction of downfolded Wannier Hamiltonians S9

C. J_{ij} Calculations S10

D. Phonon Calculations S12

References S15

S1. INELASTIC X-RAY SCATTERING

Figure S1 shows the IXS spectrum collected along $\mathbf{q} = (0, 0, l)$ at 50 K. The direction in Fig. S1 corresponds to the proposed $\mathbf{q}_{\text{nest}}$ from first principle calculations. Careful inspection between the collected spectra at 50 K and 130 K does not suggest any softening to occur. More importantly though, single crystal X-ray diffraction (XRD) suggested $\mathbf{q}_{\text{CDW}} = (1/2, 1/2, 0)$ [1]. Therefore, the main paper focuses on this latter direction/orientation. We note that the critical temperature, T_{MIT}, of 102 K obtained using IXS is slightly higher than the value of 95 K obtained from resistivity measurements [2]. The recently synthesized $K_2Cr_8O_{16}$ has $T_{\text{MIT}} \simeq 100$ K, slightly higher than the originally reported $T_{\text{MIT}} = 95$ K. This difference is attributed to an improved sample synthesis protocol (see methods section in main text). This is evidenced from the larger crystal that can be obtained, and the reduced amount of impurity phase in powder sample.

There is a small yet apparent shift of the elastic peak in Fig. 4(a) in the main text. The instrumental accuracy is expected to be $\sim$ 0.2 meV but the shift is about 0.45 meV. This shift is most likely induced by X-rays scattered from the instrumental setup, *e.g.* Be window. This is confirmed by the temperature dependence of the inelastic peak observed around 10 meV, as shown in Fig. 4(a) of the main text. Figure S2 shows this peak position as a

function of temperature. The peak position at 115 K is not shifted +0.45 meV compared with the low temperature counterparts, as one would expect if the elastic peak shift was sample contribution. Instead, the 115 K peak position is slightly lower than the low temperature one. Therefore, the elastic peak shift can be concluded to be contributions from the instrument.

S2. NEUTRON SCATTERING

Neutron diffraction patterns and the corresponding Rietveld refinement at each state (paramagnetic (PM), ferromagnetic (FM) metal and FM insulator) of the sample are shown in Fig. S3. Vanadium peaks (the sample holder) together with a small impurity phase of about 2 % CrO_2 can be observed in the pattern, as also found in other studies [2, 3]. Such phases were excluded and/or omitted from the refinement without affecting the refinement results. However, any structural monoclinic distortion were not observed below T_{MIT} within the current experimental resolution. It should be noted that the measurement was performed on a polycrystalline sample and such distortion has only been seen in single crystal measurements [1, 2], due the weak intensity of the superstructure peaks. Similar to the laboratory XRD results, no anomaly or significant change in the lattice parameter is observed around the MIT.

A. Magnetic structure

At high temperatures, the pattern can be satisfactory refined using the given crystal solution of $I4/m$ (#87) with a resulting $\chi^2 = 4.55$ and an R_F factor of 3.692 [Fig. S3(a)]. Upon cooling, a strong enhancement in a peak is observed below 167 K, consistent with the onset of a FM order. A clear change in intensity is seen for the {1,2,1} peaks as the temperature is lowered (Fig. S3), indexed according to the tetragonal structure. It is noted that the notation {1,2,1} specifies all equivalent planes. The change in intensity confirms a commensurate FM phase with $\boldsymbol{k} = (0, 0, 0)$. By using symmetry arguments, we first establish the magnetic structure in the FM state above T_{MIT}. Using the given $\boldsymbol{k}$ and the parent structure, several symmetrically allowed solutions are proposed from the Bilbao crystallographic server [4–7]. Since a FM solution is sought, most of the suggested solutions can be disregarded, as FM is forbidden under such symmetries. The highest subgroup among

the suggested ones with an allowed FM solution is the $I4/m$ (#87.75) with the irreducible representation mGM1+, in Shubnikov notation. However, the resulting ordered moment is about 1.9 μ_B, which is lower than the expected value of about 2.25 μ_B [2]. Moreover, the magnetic peak is not fully reproduced by the model, resulting in a relatively high $\chi^2 \sim 100$. The second highest subgroup that allows a FM order is the $C2'/m'$ (#12.62), described by the irreducible representations mGM$_3^+$GM$_4^+$ in Shubnikov notation resulting in $\chi^2 = 13$ and R_F factor of 5.56. The given solution allows for two magnetically unique Cr sites, each with distinct values of moments. However, the refinement is performed by assuming the moments of both Cr to be equal, since the sample is known to be FM. For the sake of completeness, we wish to mention that an even lower solution, the Cm' (#8.34) is a viable candidate as well. However, the overall refinement quality do not change compared to the $C2'/m'$ (#12.62) solution. Therefore, $C2'/m'$ (#12.62) is set to be the solution for $K_2Cr_8O_{16}$ from the NPD data.

Given the Shubnikov group, the magnetic structure can be determined. Unfortunately, a clear FM contribution is only visible at peaks {1,2,1}, which splits into 8 equivalent peaks for $\boldsymbol{k} = (0, 0, 0)$. Since only the perpendicular component of the magnetisation can be detected, the powder average is effectively averaging the magnetic components within the {x,y,0} planes. In other words, a solution $(x, 0, 0)$, $(0, y, 0)$ or $(x, y, 0)$ cannot be uniquely solved in this case. A magnetic component in the z-direction is forbidden by symmetry for the magnetic space group solution obtained in the metallic phase.

Single crystal neutron diffraction (ND) was performed to resolve in the direction of the ordered moment within the ab plane.. High statistic datasets with a collection of peaks for single crystal refinement were collected at 5 K, 130 K and 200 K, corresponding to FM insulator, FM metal and PM phases, respectively. These data sets were refined using the magnetic structure determined from powder measurements. The refinement does not yield a significant difference between the two phases. However, the orientation within the ab plane is solved uniquely in which the moments are aligned $-45°$ with respect to the b-axis. On the other hand, FM compounds are known to form magnetic domains. Including domain degree of freedom in the refinement results in an ambiguous orientation of the moments. Therefore, similar to in powder case, the ordered moment cannot be uniquely resolved within the ab plane.

B. Magnetic excitations

The inelastic neutron scattering spectra collected at 5, 130 and 200 K are shown in Fig. S3 D for the incident energies $E_i = 9$ meV and $E_i = 97$ meV. The Bose factor was divided out from the spectra in order to directly compare the spectral features at different temperatures. The white regions represents uncaptured momentum and energy transfers regions for the given instrumental configuration. The overall dispersion intensity increases as the temperature is lowered, supporting a magnetic origin. The unknown dispersion-like feature manifested at all temperatures for $E_i = 9$ meV between $Q = 1 - 2.6$ Å^{-1} for $E = 0 - 2$ meV. While its intensity is temperature dependent, the "dispersion" in itself is not and is also present at 200 K. This asymmetric "dispersion" was not reproduced by DFT or linear spin wave theory and is most likely not a contribution from the sample. Its origin is currently unknown.

Figure S3 D(d-f) show the dispersion relation collected at 5, 130 and 200 K with an incident energy of 97 meV. For these spectra, the phonon contributions are estimated from a high Q cut ($= 8.5$ Å^{-1}), which is extrapolated to lower Q by considering the phonon form factor. This method effectively subtracts optical phonons but not acoustic ones. A dispersion emerges from $Q = 2.6$ Å^{-1}, consistent with the observed magnetic peak at {2,1,1} in Fig. S3. Naturally, the acoustic phonon branch overlaps with magnons, given that the sample is a FM. However, most of the signal emerging from $Q = 2.6$ Å^{-1} can be considered to be magnetic in origin, assuming that the intensity scales with the {2,1,1} Bragg peak ($Q = 2.6$ Å^{-1}) in each phase, which show a small nuclear but large magnetic contributions. The excitations at 200 K on the other hand may be attributed to magnetic correlations remaining even above T_C, due to the low dimensional nature of the system. Additionally, a region of higher intensity is observed around 60 meV at low Q. Its Q dependence follows the one expected for a magnetic form factor, and its intensity increases with decreasing temperature. Therefore, this feature is assumed to be magnetic in origin.

The inelastic neutron scattering spectra were simulated using linear spin wave calculations performed with SpinW [8]. The agreement between the measurements and the spin wave calculations was not improved by adding a single-ion anisotropy in the Hamiltonian. Moreover, any excitation gap was not observed within the current experimental setup. The calculated spectra are also convoluted with a normal distribution given by the instrumental

energy resolution.

The exchange parameters obtained from the fits are summarized in the main text. The calculated spectra based on these parameters reproduce the experimental data well, capturing both the flat band like feature around 55 meV and the emerging dispersion near 2.6 Å^{-1} (Fig. S3 D(g,h)). This confirms that the dominant magnetic units are groups of four corner-sharing chains, as described in the main text. The consistent match between experiment and theory over the full energy range demonstrates that the extracted parameters are robust and physically meaningful.

The confidence in the obtained fit parameters is implied from Fig. S5. J_2 needs to be relatively small in order to reproduce the depletion region shown with dotted black lines in Fig. S3 D(a,b). Such feature is particularly highlighted for a cut at constant $Q = 0.6$ Å^{-1} and is shown in Fig. S5. Spin wave calculations demonstrate that the value of J_2 steadily shift the cutoff of the depleted region to higher energy transfers. Naturally, such region is not present at 200 K, as expected for a PM sample. Notably, the experimental values of this cutoff is different for 5 and 130 K, as also seen directly in Fig. S3 D(a,b). Based on Fig. S5, small values of J_2 can thus be expected, around $0.3 - 0.7$ meV for 5 K, consistent with the obtained fit values presented in the main text. It is noted that varying J_1 and J_3 does not affect the dispersion significantly in this cut.

Several Q and energy cuts (as highlighted in Fig. S3 D(a,b)) are presented in Fig. S6, showcasing good agreement between fitted and measured data. The calculation slightly underestimates the intensity, which is likely caused by a non-linear background and the presence of a much stronger peak as $Q \to 0$ Å^{-1}. In addition, there are spurious features at lower energies, a feature which is not accounted for by the model.

S3. THEORETICAL METHODS

A. DFT Calculations

The density-functional theory (DFT) calculations were performed using the `Quantum Espresso` [9] package, which uses a plane-wave basis set. The generalized gradient approximation (GGA-PBE) [10] was used for the electron exchange and correlation energy. All first-principles calculations (band structures, J_{ij}, phonon and Weyl points analyses) were

performed in the ferromagnetic spin-polarized state, for both the insulating and metallic phases. For noncollinear calculations that include spin-orbit coupling (SOC), fully relativistic (FR) pseudopotentials [11, 12] were used. When SOC was neglected, scalar-relativistic pseudopotentials for K [13, 14], Cr [15, 16] and O [14, 17] were used instead. The plane-wave cutoff for the DFT calculation was set to 70 Ry and 40 Ry for the plane-wave expansion of the wavefunctions of the fully relativistic and scalar relativistic calculations, respectively. For the self-consistent DFT calculations, the primitive unit cells of the tetragonal and monoclinic crystal structures were used, with corresponding **k**-grids of $10 \times 10 \times 4$ and $2 \times 2 \times 8$, respectively. For the monoclinic structure, the on-site Coulomb energy (also known as the Hubbard-U correction) of $U = 4.0$ eV was applied within rotationally invariant formulation [18, 19], using the Löwdin-orthogonalized atomic $3d$ pseudowavefunctions of the Cr atoms as the localized basis set for the Hubbard manifold. In contrast, for the metallic tetragonal phase, no Hubbard correction was applied (i.e., $U = 0$ eV). While calculations with $U = 0$ eV reproduce the experimental trends, we cannot exclude the possibility that on-site corrections that include dynamical or multi-configurational effects may be needed to reproduce experimental magnitudes. Such effects would require approaches beyond DFT+U, such as dynamical mean-field theory (DMFT), which goes beyond the scope of this work.

The DFT band structure calculations were calculated based on structural information derived from experiments. We started with the experimentally derived atomic structure [20] and proceeded to relax the lattice structure and the atomic positions. This was accomplished by minimizing every calculated force component on the ions, the total energy of the structure and the pressure within the crystal, while neglecting perturbative effects due to spin-orbit coupling. The minimization procedure is considered converged when every calculated force component on the ions, the total energy of the structure and the pressure between the last two relaxation iterations are converged to within 10^{-8} Ry/bohr, 10^{-15} Ry and 0.1 kbar, respectively. The relaxed lattice parameters we obtained were $a = b = 26.24$ bohr, and $c = 5.59$ bohr. Similarly, we start from the experimental lattice and atomic positions for the monoclinic structure [1], relaxing only the atomic positions, which were subsequently used to calculate the phonon band structure.

Noncollinear DFT+SOC calculations were conducted to account for the ferromagnetic ordering and to study the Weyl points. In the conventional tetragonal cell, each Cr atom carries a net spin moment of 1.9 μ_{B}, all aligned along the same direction. The azimuthal

orientation of the spins (ϕ, measured in the xy-plane from the x-axis toward the y-axis) varies only slightly, with a standard deviation of 0.004°.

To directly compare with the insulating phase, we also performed calculations using a monoclinic lattice supercell, equivalent to the enlarged face-centered conventional tetragonal unit cell. These calculations were performed for several FM moment directions (Fig. S8). In the monoclinic supercell, modulations of the wavefunction periodicity slightly break the fractional translational symmetry that relates it to the tetragonal cell. While the net Cr moment remains 1.9 μ_B, inclusion of spin-orbit coupling introduces a slight canting, increasing the standard deviation of ϕ to 0.01°. This induces subtle energy modulations of the nearly flat bands in which the Weyl points reside, shifting them from their original positions and relocating those identified in the primitive and conventional tetragonal cells onto nodal planes of the monoclinic supercell. At the same time, the folded bands develop small gaps at the Brillouin-zone boundary. Together, these effects explain the differences between the Weyl point positions reported here and those in earlier studies [21].

Due to the resulting high density of states near the Fermi level originating from the symmetry-enforced nodal plane, $k_c = \pm\pi/c$, the band manifold is highly sensitive to perturbations, like strains, which can weaken the screw and translational symmetries. Therefore, the exact positions of the Weyl points are sensitive to the lattice and atomic positions (even though their general positions forming a cross on the $k_c = \pi/c$-plane are not). To ensure the reproducibility of our calculations, we provide in Table S1 the atomic positions of the tetragonal phase we used, that are obtained from our neutron diffraction experiments.

In Fig. 3(c,d) of main text, the **k**-paths of the bandstructures were labelled using special **k**-points of the $I4/m$ space group, as listed in Table S2. However, since the calculated DFT eigenenergies are not quasiparticle energies due to the lack of quasiparticle self-energy correction, e.g., via the GW correction, we rigidly increased the bandgap of the monoclinic phase by 0.12 eV to reproduce the experimental bandgap of 40 meV at base temperature. The Fermi surface of the metallic phase was also calculated non-self-consistently on a $28 \times 28 \times 12$ **k**-grid for its primitive unit cell and plotted in Fig. S7, in agreement with literature [22].

B. Construction of downfolded Wannier Hamiltonians

In order to calculate the magnetic exchange parameters, J_{ij}, and the chiralities of the Weyl points (Table S3), tight binding models in the basis sets of maximally localized Wannier functions [23, 24] were constructed using `Wannier90` [25]. In this work, the spatial orientation of our Wannier d-orbitals are defined with respect to the *local* z-axis of the octahedron and not the *global* z-axis of the crystal lattice. We define the origin of the axes to be at the central Cr atom. The z-axis of the octahedron to be pointing along the shortest Cr-O bonds. The y-axis is defined as the vector that points along the mirror plane and then rotated 45° in-plane (see Fig. 3(a) in Main Text). Due to the crystal symmetry, z-axis of the octahedron, always lies in the ab-plane. Since each Cr-O octahedron is distorted (i.e., their Cartesian x-, y-, and z-axes along the Cr-O bonds are not orthogonal), we define our orthogonal axes that were used to define the d-orbitals by gently perturbing the non-orthogonal axes away from the bond directions in a way that maximizes the sum of their dot products with our orthogonal axes.

For the Weyl-point calculations, the Wannier Hamitonians of both the tetragonal and monoclinic phases were constructed in the monoclinic lattice supercell using DFT wavefunctions calculated non-consistently on a $4 \times 4 \times 8$ $\mathbf{k}$-grid. Using the Wannier tight-binding models, we searched for chiral nodes using `WannierTools` [26] on an interpolated $32 \times 32 \times 96$ $\mathbf{k}$-grid. For the metallic phase, the Wannier basis states consisted of only the t_{2g}-likes states of Cr, since they form an isolated manifold of Bloch bands just below the Fermi level. We found that other than the slight increase in the bandwidths, the qualitative features of electronic band structure remains largely the same (Fig. S9). For our analysis we use $U = 0$ eV. For the insulating phase, the Hubbard-U increases the energy separation between the unoccupied e_g-likes states (which increases in energy) and the partially occupied t_{2g}-likes states (which decreases in energy), such that the latter merges with the otherwise isolated O $2p$-manifold just below it. Consequently, the Wannier basis states consisted of not only the Cr t_{2g}-likes states, but also the O $2p$-states. The calculated Weyl points are listed in Table S3.

C. J_{ij} Calculations

For each J_{ij}, contributions from all orbitals at each site have been summed. Here, i and j are the site indices. Each site contains one or more orbitals. Without the inclusion of O Wannier functions in the Wannier basis set, the tight-binding band structures interpolated from the Cr t_{2g}-like Wannier functions (with partial O characters) reproduce the low-energy disentangled [24] DFT bands very well for $U = 0.0$ to 2.5 eV. As U increases above 2.5 eV, the Wannier-interpolated band structures begin to deviates away from the DFT bandstructures, but were used when performing systematic comparisons of the J_{ij} between the hypothetical low-U calculation of the monoclinic phase and the $U = 0$ eV calculation of the tetragonal phase. In order to calculate the magnetic exchange interaction, we use the magnetic force theorem [27, 28] to map the tight-binding Wannier Hamiltonian onto a classical Heisenberg model (as defined in the Main Text) using `TB2J` [29].

In agreement with our neutron scattering experiment (Sec. II of Main Text), the theoretically calculated exchange parameters are also smaller between the nearest neighbors, J_1 and J_2, than they are for the third exchange parameter, J_3 (Fig. S10(c - top panel)). From a chemical perspective, the results can be understood from the fact that the nearest neighboring Cr atoms are bridged by an intermediate O atom at an angle close to 90° (at $\sim 97°$). According to the Goodenough-Kanamori rules, the near-90° bond angles between nearest neighbors favor ferromagnetic exchange but suppress the strength of orbital overlap. On the other hand, the third-nearest neighbors are bridged by an intermediate O atom at $\sim 130°$, resulting in the overlap integral between the third-nearest neighbors is larger than the overlap between the nearest neighbors. This is verified by our DFT calculations and Wannier Hamiltonian, which show that the former has larger hopping magnitudes (Fig. S10(c)) (see Sec. S3 of SM for more information). Therefore, the system does not experience quantum confinement effects expected of a strictly 1D system, even though $K_2Cr_8O_{16}$ is generally considered quasi-1D. This is also consistent with the absence of a Peirels instability: since the 1D-building block is a chimney instead of a linear chain, electronic motions in $K_2Cr_8O_{16}$ have an additional degree of freedom and cannot be considered strictly 1D (Fig. 1 of Main Text). Consequently, the electronic (and magnetic) response does not diverge.

In order to elucidate the exchange mechanisms across the phase transition, we present the calculated exchange integral, J, as a function of the calculated hopping integral, t, and the

Coulomb (Hubbard) matrix element, U (Fig. S10). A clear t^2/U dependence is found, indicating that the average interactions in $K_2Cr_8O_{16}$ can be described within a superexchange framework [30, 31] for both the insulating (Fig. S10(a)) and metallic (Fig. S10(b)) phases. Interestingly, this quadratic dependence is not characteristic of the double exchange (DE) mechanism [22, 32–34], for which $J_{\mathrm{DE}} \propto t$ [30, 31]. Even though K_2Cr8O_{16} may nominally be regarded as a mixed-valence compound of the form $K_2[Cr^{3+}]_2[Cr^{4+}]_6O_{16}$, where DE is usually expected especially in the FM metallic phase, the DE picture is anomalous for the insulating phase. In fact, DFT calculations show that all Cr ions possess nearly the same fractional oxidation state ($\sim +3.75$) in the metallic phase, consistent with their crystallographic equivalence and in line with the valence bond analysis of Ref. [1]. Thus, rather than electron transfer between distinct Cr^{3+} and Cr^{4+} ions, the dominant hopping occurs between partially filled isovalent Cr t_{2g} states, which is more akin to a superexchange process.

It is important to note that although the canonical t^2/U dependence is often associated with antiferromagnetic superexchange (particularly for t_{2g}–t_{2g} or e_g–e_g hopping), ferromagnetic exchange can also arise within the same second-order perturbative framework under conditions described by the Goodenough–Kanamori–Anderson (GKA) rules. In $K_2Cr_8O_{16}$, the Cr–O–Cr bond angles are close to 97°, which strongly suppresses direct $t2g$–t_{2g} overlap and favors nearly orthogonal orbital interactions. In this situation, Hund's coupling on the intermediate O $2p$ orbitals promotes ferromagnetic alignment when an electron virtually hops from a half-filled (Cr^{3+}, d^3) to an empty (Cr^{4+}, d^2) orbital. This "Hund's-rule-driven" ferromagnetic superexchange mechanism preserves the quadratic scaling with t, such that the resulting exchange still follows a t^2/Δ dependence, where Δ is an effective charge-transfer excitation energy incorporating both the oxygen site energy and Hund's exchange.

This interpretation is fully consistent with: (1) our neutron scattering results, which show that magnetic interactions are essentially unchanged across the FM–MIT; (2) inelastic x-ray scattering measurements excluding a Peierls mechanism, upon which the previously proposed insulating-state DE picture was based; and (3) the general expectation that superexchange interactions, including their ferromagnetic variants, can operate in both metallic and insulating phases of transition-metal oxides.

D. Phonon Calculations

For the calculations of the phonon dispersions for the insulating and metallic phases, we solved for the phonon eigenvectors and eigenvalues with the finite displacement and supercell approach using `Phonopy` [35]. Within the finite displacement approach, the force constants of the insulating phase were calculated using a supercell of $1 \times 1 \times 5$ and a shifted **k**-grid of $4 \times 4 \times 4$ using spin-polarized DFT but neglecting perturbative spin-orbit coupling effects. The force constants of the metallic phase were calculated using a supercell of $1 \times 1 \times 4$ and a shifted **k**-grid of $2 \times 2 \times 2$. Integration over the Brillouin zone was performed using the optimized tetrahedron method [36]. Within the implementation of `Quantum Espresso`, forces cannot be calculated using the Löwdin-orthogonalized atomic $3d$ pseudowavefunctions, hence atomic orbitals were used instead to build the Hubbard projectors for the insulating phase. With this choice of localized basis set, the bandstructure of the insulating phase is only gapped when the lattice is under a compressive strain of 0.3% in the a- and b-axes and 6.4% in the c-axis, relative to the experimental structure of Ref. [1] (see Fig. 1(a) of Main Text for the definitions of the crystallographic axes), with the atomic positions relaxed by minimizing DFT forces. The strain does not qualitatively change the bandstructure of the occupied states for the metallic phase, but is necessary to capture the physics related to the gapped nature of the electronic band structure (Fig. S11(d)). Importantly, we see that when calculating the phonon dispersion for the insulating (Fig. S11a) and metallic (Fig. S11(b)) phases using the same lattice parameters, no phonon condensation was observed. The applied strain also does not introduce qualitative changes to the phonon dispersion (Fig. S11(b) vs Fig. S11(c)), besides increasing (or renormalizing) the phonon energies when compressive strain is applied. In Fig. S11, we plotted the phonon dispersions and electronic DFT bandstructures of both the metallic and insulating phases by mapping the atomic positions of both phases into the same unit cell.

TABLE S1: Atomic positions of the metallic phase in the face-centered conventional unit cell in fractional coordinates of the lattice.

atom types	k_x	k_y	k_z
O	0.6485611636	0.1894267592	0.0000000000
O	0.3514388964	0.8105732408	0.0000000000
O	0.8514388514	0.8105732410	0.5000000000
O	0.1485611186	0.1894267590	0.5000000000
O	0.1894267592	0.3514388964	0.0000000000
O	0.8105732408	0.6485611636	0.0000000000
O	0.3105732410	0.6485611636	0.5000000000
O	0.6894267590	0.3514388964	0.5000000000
O	0.3514388964	0.3105732410	0.5000000000
O	0.6485611636	0.6894267590	0.5000000000
O	0.1485611188	0.6894267590	0.0000000000
O	0.8514388512	0.3105732410	0.0000000000
O	0.8105732410	0.1485611186	0.5000000000
O	0.1894267590	0.8514388514	0.5000000000
O	0.6894267590	0.8514388512	0.0000000000
O	0.3105732410	0.1485611188	0.0000000000
O	0.0242460598	0.3208810688	0.5000000000
O	0.9757538622	0.6791189312	0.5000000000
O	0.4757539464	0.6791189310	0.0000000000
O	0.5242461436	0.3208810690	0.0000000000
O	0.1791189316	0.5242461436	0.5000000000
O	0.8208810684	0.4757539464	0.5000000000
O	0.3208810690	0.4757539464	0.0000000000
O	0.6791189310	0.5242461436	0.0000000000
O	0.9757538624	0.1791189316	0.0000000000

Continued on next page

TABLE S1 – continued from previous page

atom type	r_1	r_2	r_3
O	0.0242460596	0.8208810684	0.0000000000
O	0.5242461436	0.8208810684	0.5000000000
O	0.4757539464	0.1791189316	0.5000000000
O	0.8208810684	0.9757538624	0.0000000000
O	0.1791189316	0.0242460596	0.0000000000
O	0.6791189312	0.0242460598	0.5000000000
O	0.3208810688	0.9757538622	0.5000000000
K	0.0000000000	0.0000000000	0.5000000000
K	0.5000000000	0.0000000000	0.0000000000
K	0.0000000000	0.5000000000	0.0000000000
K	0.5000000000	0.5000000000	0.5000000000
Cr	0.0918905380	0.2558853888	0.0000000000
Cr	0.9081095200	0.7441145812	0.0000000000
Cr	0.4081094622	0.7441145812	0.5000000000
Cr	0.5918904798	0.2558853888	0.5000000000
Cr	0.2558853888	0.4081094622	0.5000000000
Cr	0.7441145812	0.5918904798	0.5000000000
Cr	0.2441146116	0.5918904800	0.0000000000
Cr	0.7558854184	0.4081094620	0.0000000000
Cr	0.9081095202	0.2441146114	0.5000000000
Cr	0.0918905378	0.7558854186	0.5000000000
Cr	0.5918904800	0.7558854184	0.0000000000
Cr	0.4081094620	0.2441146116	0.0000000000
Cr	0.7441145812	0.0918905380	0.0000000000
Cr	0.2558853888	0.9081095200	0.0000000000
Cr	0.7558854186	0.9081095202	0.5000000000
Cr	0.2441146114	0.0918905378	0.5000000000

TABLE S2. Fractional coordinates of the special **k**-points in its reciprocal unit cell

special **k**-point	k_a	k_b	k_c
Γ	0.0	0.0	0.0
Z	0.0	0.0	0.5
D	0.5	0.0	0.5
B	0.5	0.0	0.0
A	0.5	-0.5	0.0
E	0.5	-0.5	0.5
C	0.0	-0.5	0.5
Y	0.0	-0.5	0.0

TABLE S3. Fractional coordinates of the selected Weyl points calculated for the metallic phase in a monoclinic supercell.

k_a^{M}	k_b^{M}	k_c^{M}
-0.2631	0.0275	-0.4962
-0.0083	-0.2486	0.4863
0.0033	0.2342	0.4858
-0.0056	-0.2517	0.4973
0.0169	0.2563	-0.4966
-0.0093	-0.2634	-0.4885

[1] T. Toriyama, A. Nakao, Y. Yamaki, H. Nakao, Y. Murakami, K. Hasegawa, M. Isobe, Y. Ueda, A. V. Ushakov, D. I. Khomskii, S. V. Streltsov, T. Konishi, and Y. Ohta, Phys. Rev. Lett. **107**, 266402 (2011).

[2] K. Hasegawa, M. Isobe, T. Yamauchi, H. Ueda, J.-I. Yamaura, H. Gotou, T. Yagi, H. Sato, and Y. Ueda, Phys. Rev. Lett. **103**, 146403 (2009).

[3] J. Sugiyama, H. Nozaki, M. Månsson, K. Prša, D. Andreica, A. Amato, M. Isobe, and Y. Ueda, Phys. Rev. B **85**, 214407 (2012).

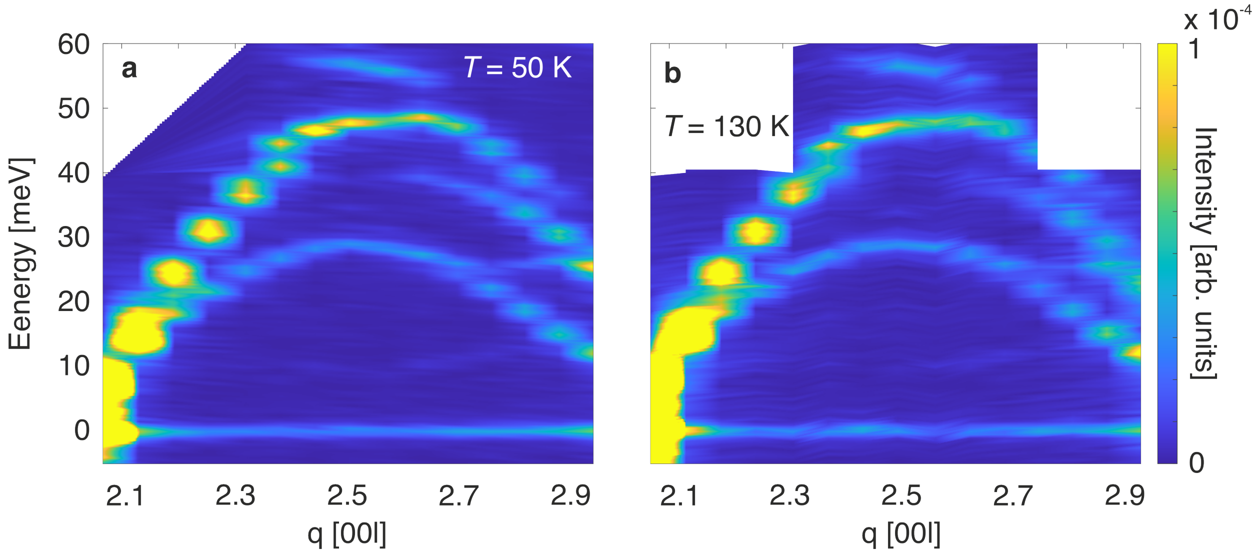

Figure S1. Phonon dispersion along $\mathbf{q} = (0, 0, l)$. Example of an interpolated inelastic X-ray scattering (IXS) spectra of $K_2Cr_8O_{16}$ collected at (a) $T = 50$ K and (b) $T = 130$ K along $\mathbf{q} = (0, 0, l)$, up to 60 meV energy transfers. Both panels use the same colour scale.

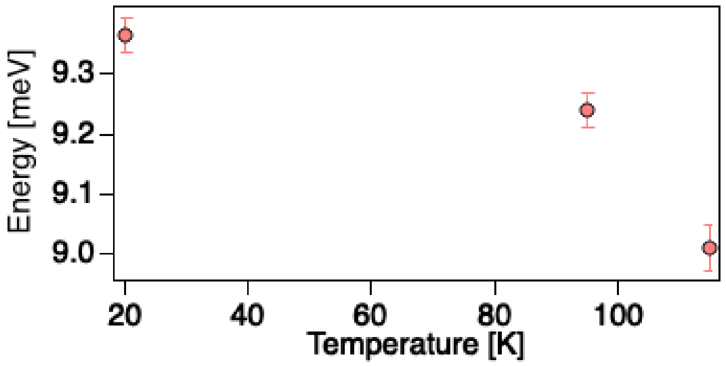

Figure S2. The inelastic peak position. The inelastic peak position in energy (from Fig. 2(a) in main text) as a function of temperature. The inelastic peak at $T = 115$ K doesn't have a similar positive shift similar to the elastic peak. Error bars represent the experimental uncertainty.

[4] M. I. Aroyo, J. M. Perez-Mato, C. Capillas, E. Kroumova, S. Ivantchev, G. Madariaga, A. Kirov, and H. Wondratschek, Zeitschrift für Kristallographie - Crystalline Materials **221**, 15 (01 Jan. 2006).

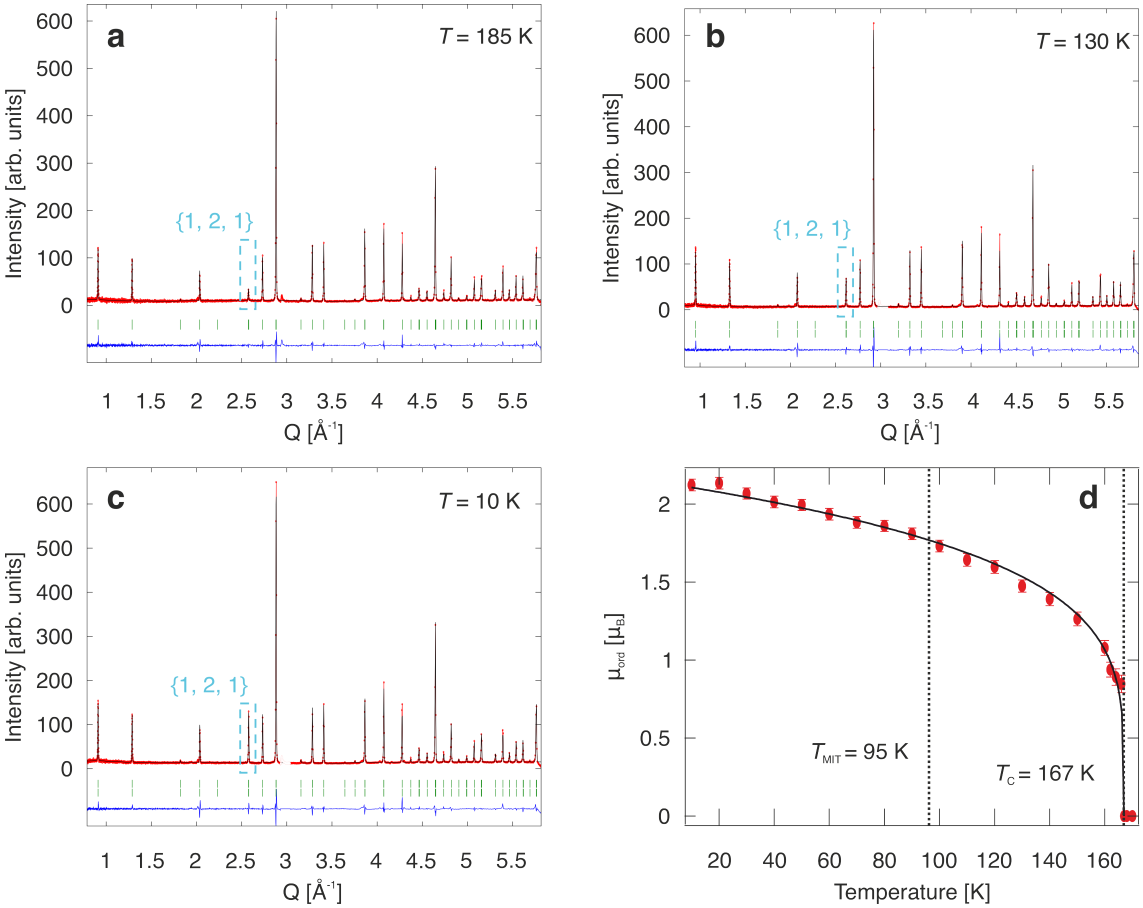

Figure S3. Powder neutron diffraction patterns. Diffraction patterns collected at (a) 185 K (paramagnetic metal), (b) 130 K (ferromagnetic metal) and (c) 10 K (ferromagnetic insulator). Experimental data are shown in red, and the best Rietveld refinement including the magnetic contribution is shown as a solid black line. The difference between the data and the refinement is shown below as a solid blue line. Allowed Bragg reflection positions are indicated by green markers. A vanadium peak at approximately $Q = 3$ Å^{-1}, arising from the sample holder, was omitted from the refinement. (d) Ordered magnetic moment of the Cr ions as a function of temperature. The solid line represents the best fit using $\mu(T) = \mu(0)(1 - T/T_{\mathrm{C}})^{\beta}$, with $\mu(0) = 2.135(19)$, $T_{\mathrm{C}} = 167.0(1)$ K, and $\beta = 0.219(7)$. Error bars represent the experimental uncertainty.

[5] M. I. Aroyo, A. Kirov, C. Capillas, J. M. Perez-Mato, and H. Wondratschek, Acta Crystallographica Section A **62**, 115 (2006).

[6] M. Aroyo, J. Perez-Mato, D. Orobengoa, E. Tasci, G. De La Flor, and A. Kirov, Bulgarian Chemical Communications **43**, 183 (2011), cited By 271.

[7] J. Perez-Mato, S. Gallego, E. Tasci, L. Elcoro, G. de la Flor, and M. Aroyo, Annual Review of Materials Research **45**, 217 (2015), https://doi.org/10.1146/annurev-matsci-070214-021008.

[8] S. Toth and B. Lake, Journal of Physics: Condensed Matter **27**, 166002 (2015).

[9] P. Giannozzi, S. Baroni, N. Bonini, M. Calandra, R. Car, C. Cavazzoni, D. Ceresoli, G. L. Chiarotti, m. Cococcioni, I. Dabo, A. D. Corso, S. Fabris, G. Fratesi, S. de Gironcoli, R. Gebauer, U. Gerstmann, C. Gougoussis, A. Kokalj, M. Lazzeri, L. Martin-Samos, N. Marzari, F. Mauri, R. Mazzarello, S. Paolini, A. Pasquarello, L. Paulatto, C. Sbraccia, S. Scandolo, G. Sclauzero, A. P. Seitsonen, A. Smogunov, P. Umari, and R. M. Wentzcovitch, J. Phys.: Condens. Matter **21**, 395502 (2009), arXiv:0906.2569.

[10] J. P. Perdew, K. Burke, and M. Ernzerhof, Phys. Rev. Lett. **77**, 3865 (1996).

[11] D. R. Hamann, Physical Review B - Condensed Matter and Materials Physics **88**, 1 (2013).

[12] M. J. van Setten, M. Giantomassi, E. Bousquet, M. J. Verstraete, D. R. Hamann, X. Gonze, and G. M. Rignanese, Computer Physics Communications **226**, 39 (2018), arXiv:1710.10138.

[13] A. Dal Corso, Computational Materials Science **95**, 337 (2014).

[14] P. Blochl, Physical review. B, Condensed matter **50** (2003).

[15] K. F. Garrity, J. W. Bennett, K. M. Rabe, and D. Vanderbilt, Computational Materials Science **81**, 446 (2014).

[16] D. Vanderbilt, Phys. Rev. B **41**, 7892 (1990).

[17] E. Kucukbenli, M. Monni, B. I. Adetunji, X. Ge, G. A. Adebayo, N. Marzari, S. de Gironcoli, and A. D. Corso, (2014), 10.48550/arxiv.1404.3015, arXiv:1404.3015.

[18] V. I. Anisimov, J. Zaanen, and O. K. Andersen, Physical Review B **44**, 943 (1991).

[19] V. I. Anisimov, F. Aryasetiawan, and A. I. Lichtenstein, Journal of Physics Condensed Matter **9**, 767 (1997).

[20] O. Tamada, N. Yamamoto, T. Mori, and T. Endo, Journal of Solid State Chemistry **126**, 1 (1996).

[21] J. Z. Zhao, Y. J. Jin, R. Wang, B. W. Xia, and H. Xu, New Journal of Physics **22** (2020), 10.1088/1367-2630/ab9d56.

[22] M. Sakamaki, T. Konishi, and Y. Ohta, Phys. Rev. B **80**, 024416 (2009).

[23] N. Marzari and D. Vanderbilt, Physical Review B **56**, 12847 (1997), arXiv:9707145 [cond-mat].

[24] I. Souza, N. Marzari, and D. Vanderbilt, Physical Review B **65**, 035109 (2001), arXiv:0108084 [cond-mat].

[25] G. Pizzi, V. Vitale, R. Arita, S. Blügel, F. Freimuth, G. Géranton, M. Gibertini, D. Gresch, C. Johnson, T. Koretsune, J. Ibañez-Azpiroz, H. Lee, J.-M. Lihm, D. Marchand, A. Marrazzo, Y. Mokrousov, J. I. Mustafa, Y. Nohara, Y. Nomura, L. Paulatto, S. Poncé, T. Ponweiser, J. Qiao, F. Thöle, S. S. Tsirkin, M. Wierzbowska, N. Marzari, D. Vanderbilt, I. Souza, A. A. Mostofi, and J. R. Yates, Journal of Physics: Condensed Matter **32**, 165902 (2020).

[26] Q. Wu, S. Zhang, H.-F. Song, M. Troyer, and A. A. Soluyanov, Computer Physics Communications **224**, 405 (2018).

[27] A. I. Liechtenstein, M. I. Katsnelson, and V. A. Gubanov, Journal of Physics F: Metal Physics **14**, 5 (1984).

[28] A. I. Liechtenstein, M. I. Katsnelson, V. P. Antropov, and V. A. Gubanov, Journal of Magnetism and Magnetic Materials **67**, 65 (1987).

[29] X. He, N. Helbig, M. J. Verstraete, and E. Bousquet, Computer Physics Communications **264** (2021), 10.1016/j.cpc.2021.107938, arXiv:2009.01910.

[30] E. Koch, Correlated electrons: from models to materials **2**, 1 (2012).

[31] Y. O. Kvashnin, R. Cardias, A. Szilva, I. Di Marco, M. I. Katsnelson, A. I. Lichtenstein, L. Nordström, A. B. Klautau, and O. Eriksson, Physical Review Letters **116**, 1 (2016), arXiv:1510.01872.

[32] M. Sakamaki, T. Konishi, and Y. Ohta, Phys. Rev. B **82**, 099903 (2010).

[33] A. Nakao, Y. Yamaki, H. Nakao, Y. Murakami, K. Hasegawa, M. Isobe, and Y. Ueda, Journal of the Physical Society of Japan **81**, 054710 (2012).

[34] S. Nishimoto and Y. Ohta, Physical Review Letters **109**, 1 (2012).

[35] A. Togo and I. Tanaka, Scr. Mater. **108**, 1 (2015).

[36] M. Kawamura, Y. Gohda, and S. Tsuneyuki, Phys. Rev. B **89**, 094515 (2014).

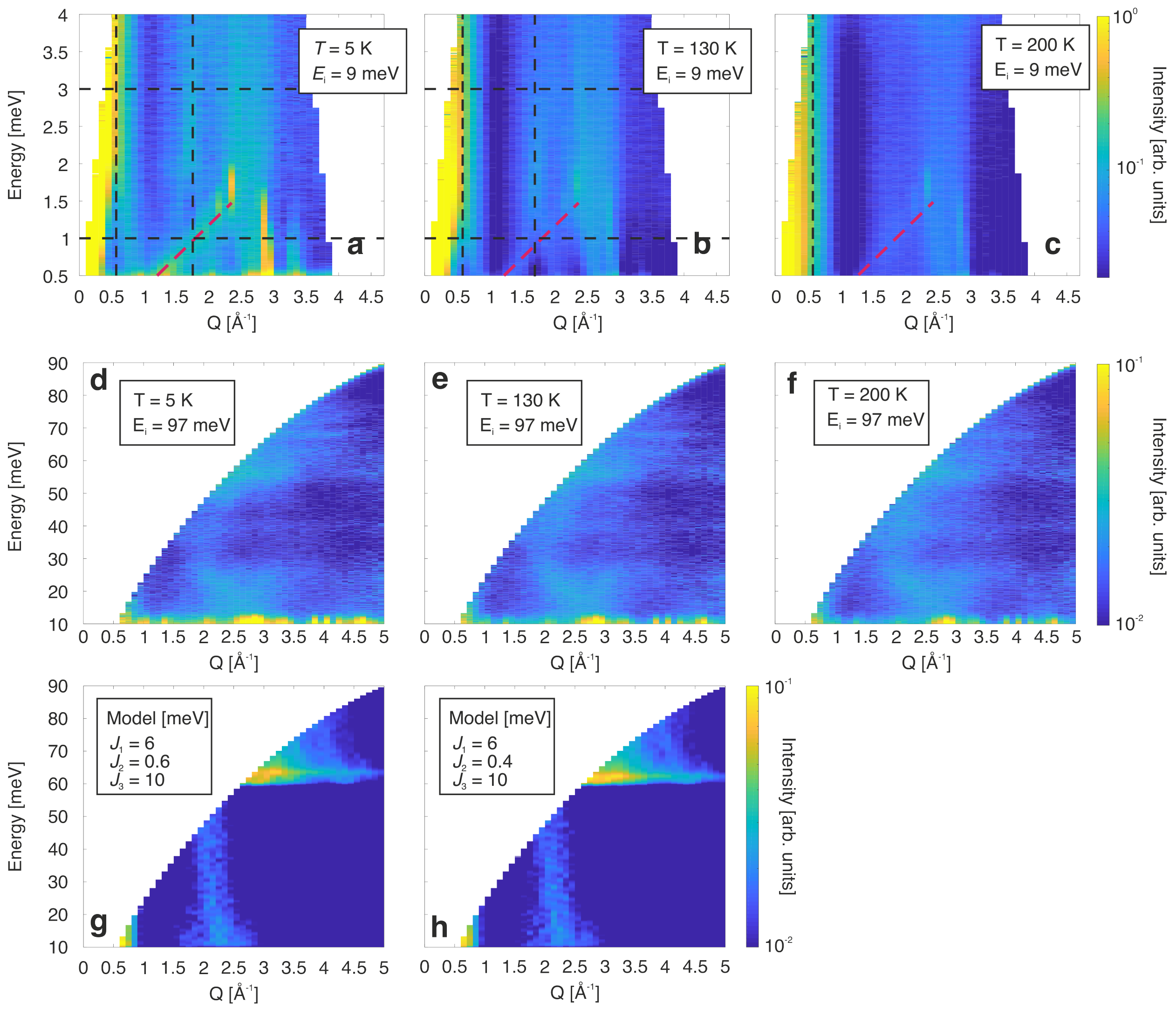

Inelastic neutron scattering spectra. Inelastic neutron scattering spectra collected at 5 K (a,d), 130 K (b,e) and 200 K (c,f) for incident energies of 9(a-c) and 97(d-f) meV. The red dashed lines in (a-c) highlight the spurious-unknown feature discussed in the text. The Bose factor was divided out from the spectra to allow a direct comparison of the spectral features at different temperatures. The dashed black lines indicate cuts presented in Fig. S5 and Fig. S6. (g,h) Calculated inelastic neutron scattering spectra obtained from the best-fit Heisenberg Hamiltonian for 5 K and 130 K, as defined in the main text. The colour scale of each figure is defined in the colour bar at the end of each row.

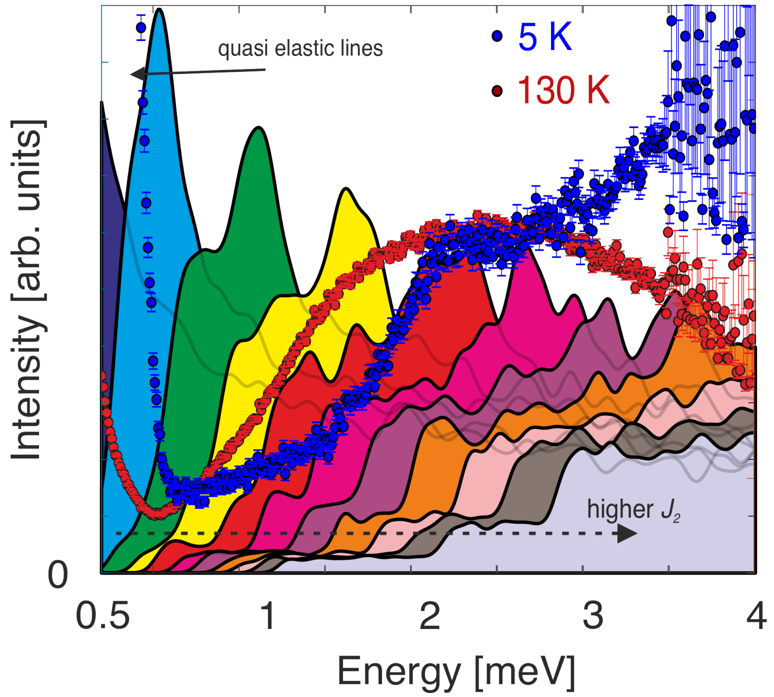

Figure S5. A cut of the inelastic neutron scattering data. A $Q = 0.6(1)$ Å^{-1} cut of the data shown in Fig. S3 D(a,b), collected at $E_i = 9$ meV, for temperatures of 5 K and 130 K (see legend). The curves correspond to experimental data at the two temperatures. The solid curves and shaded regions indicate calculated dispersion relations for $J_1 = 6$ meV and $J_3 = 10$ meV as a function of J_2, varied from 0 to 1 meV in steps of 0.1 meV. The increase in intensity at lower energies arises from quasi-elastic contributions. The excitation shows a strong dependence on J_2, with higher values shifting the excitation to higher energies. Error bars represent the experimental uncertainty.

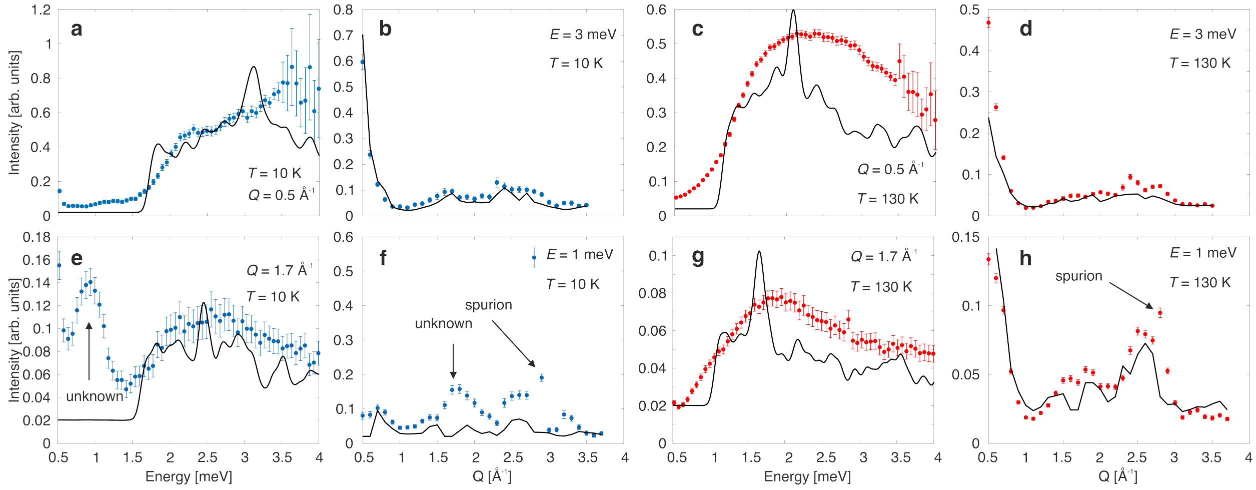

Figure S6. Inelastic neutron scattering cuts. (a–h) Inelastic neutron scattering data (symbols) and calculated magnon dispersions (solid lines) for selected constant-energy and constant-Q cuts at 10 K (a,b,e,f) and 130 K (c,d,g,h). The corresponding energy and momentum values, indicated in the panels, are marked by dashed lines in Fig. S3 D. The peak labelled as “unknown” corresponds to an asymmetric feature that is not reproduced by density functional theory or linear spin-wave calculations and is indicated by a dashed line in Fig. S3 D. Error bars represent the experimental uncertainty.

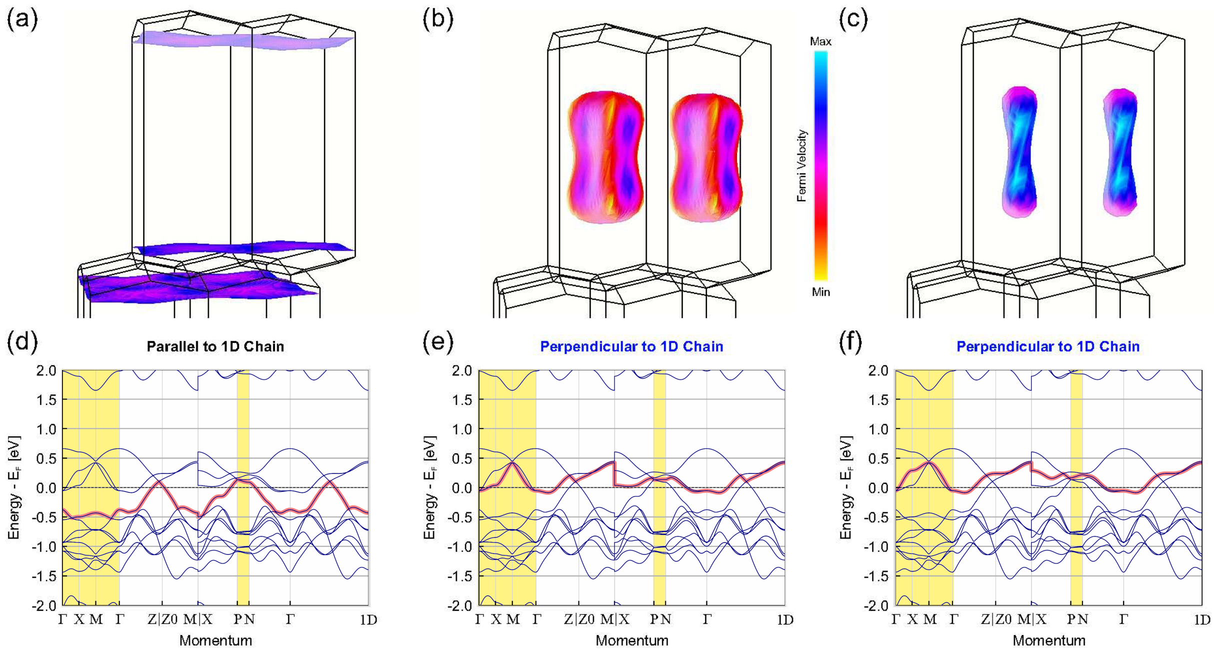

Figure S7. Calculated Fermi surface.(a-c) Calculated Fermi surface with (d-f) the corresponding calculated DFT bandstructure. The Fermi surface can roughly be divided into two compositions: (a) flat Fermi sheets originating from contributions parallel to the 1D chains (along c-axis) and (b, c) quasi 3D features originating from contributions perpendicular to the 1D chains. In (a), a strongly nested feature is observed along $\mathbf{q} = (0, 0, l)$, consistent with previously calculated surfaces [1]. This study and single crystal X-ray diffraction suggest $\mathbf{q}_{\mathrm{CDW}} = (1/2, 1/2, 0)$ [1], for which the calculated Fermi surface shows absent or weak nesting features (b,c). For each Fermi surface, the corresponding band dispersion in the bandstructure is highlighted in red in (d-f).

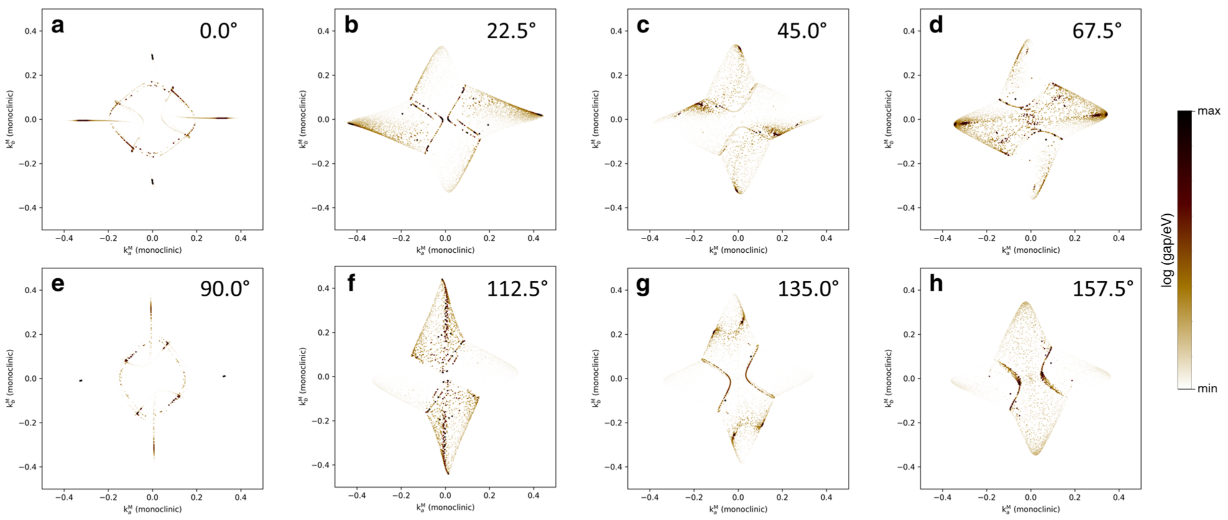

Figure S8. Cross-like nodal points and their dependence on the directions of the in-plane magnetic moment. (a–h) Evolution of the nodal points (gapless points around the $k_c = \pm\pi/c$ plane) as a function of the magnetization direction $U = 0.0$ eV). Here, the angle defines the direction of the magnetic moment relative to the a_{M}-lattice vector of the monoclinic supercell, which increases counterclockwise within the $a_{\mathrm{M}}b_{\mathrm{M}}$-plane (as defined in Fig. 1(a) of Main Text). Although changing the direction of the magnetic moment shifts the nodal lines and node positions, the overall cross-like feature remains, ensuring that nested nodal pairs persist along $[0.5, 0.0, 0.0]$ and $[0.0, 0.5, 0.0]$ in the monoclinic structure BZ (corresponding to $[0.5, -0.5, 0.0]$ and $[0.5, 0.5, 0.0]$ in the tetragonal structure BZ). Circle radii at the nodal points are proportional to log(gap), while color intensity scales as $[\log(\mathrm{gap})]^2$, highlighting regions with smaller band gaps. All panels use the same colour scale.

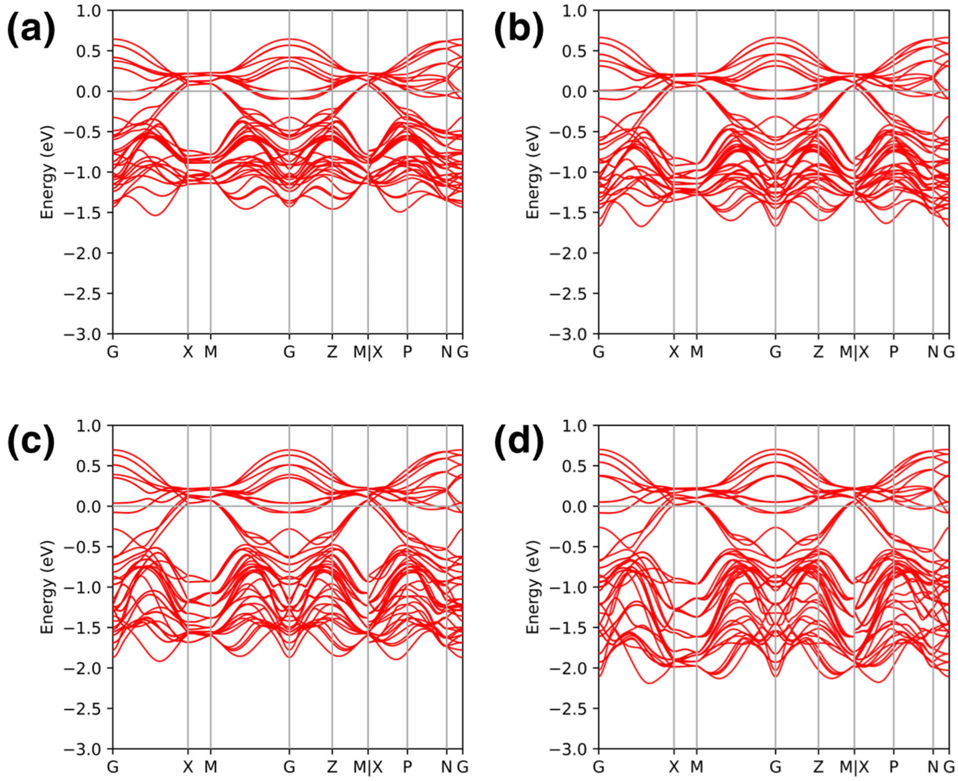

Figure S9. Wannier based band structures of the metallic phase. Calculated band structures for (a) $U = 0$ eV, (b) $U = 1$ eV, (c) $U = 2$ eV, and (d) $U = 3$ eV.

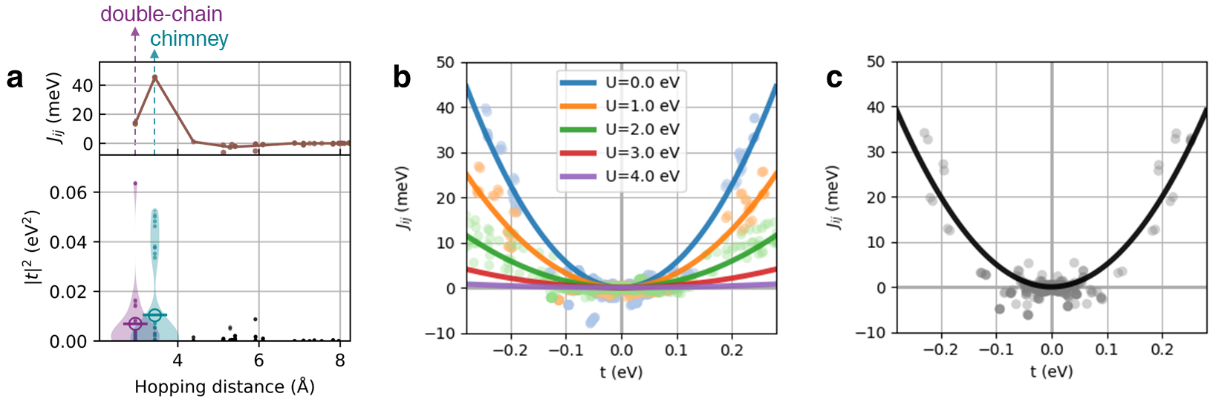

Figure S10. J_{ij} calculations. (a) Calculated J_{ij} and $|t|^2$ for the tetragonal phase. Top: Calculated J_{ij} as a function of hopping distance. Solid line passes through average J_{ij} values at each hopping distance. Bottom: Calculated $|t|^2$ as a function of hopping distance between orbitals. Hoppings between the nearest-neighboring Cr (inter-chimney) and between the next-nearest-neighboring Cr (intra-chimney) are plotted. Mean $|t|^2$ is marked using a hollow circle and a horizontal dash, and the density plot of overlapping data points are superposed. (b) Calculated J_{ij}-vs-t plots for the monoclinic phase. Calculated hoppings, t, are plotted and fitted to parabolic curves. (c) Calculated and fitted J_{ij}-vs-t for the metallic tetragonal phase (for which $U = 0.0$ eV).

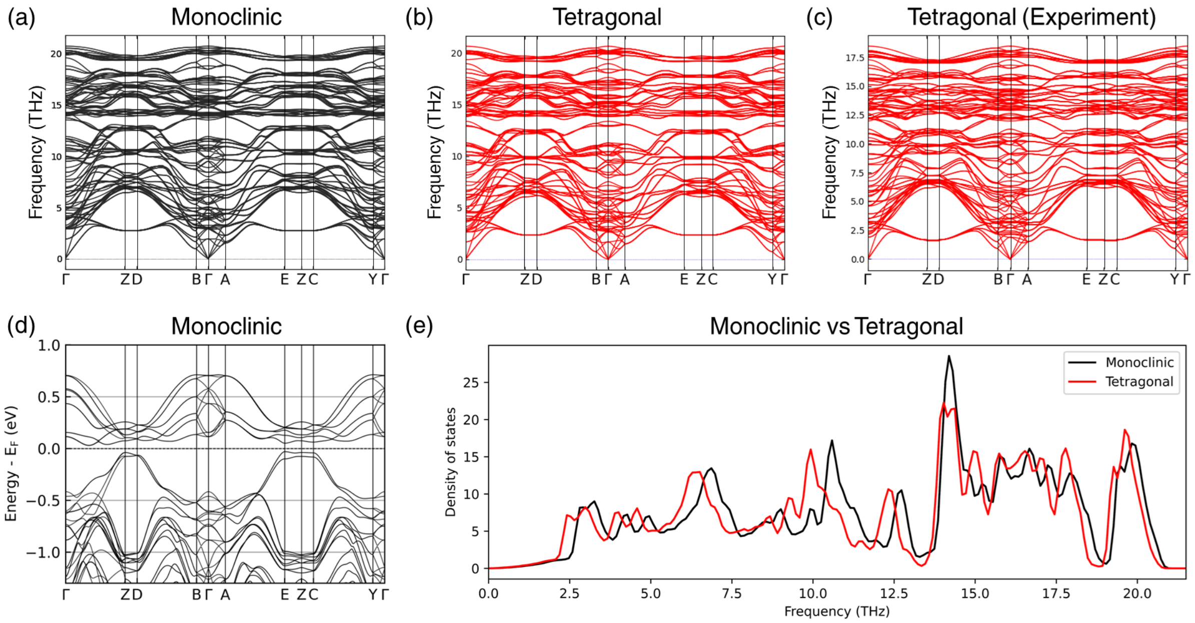

Figure S11. Calculated electron and phonon dispersions. Phonon dispersions of the (a) insulating and (b) metallic phase. (c) The phonon dispersion of the metallic tetraongal phase calculated using experimental crystal structures [1, 20]. (d) The corresponding calculated DFT bandstructures for (a). (e) The densities of states of the insulating phase (a) and the metallic phase (b).
